# Complete Genome Sequence of Stenotrophomonas maltophilia Podophage Pepon

**DOI:** 10.1128/mra.00158-22

**Published:** 2022-04-25

**Authors:** Jin Lee, Johnathan Lo, James Clark, Tram Le, Ben Burrowes, Mei Liu

**Affiliations:** a Department of Biochemistry and Biophysics, Texas A&M University, College Station, Texas, USA; b Center for Phage Technology, Texas A&M University, College Station, Texas, USA; c BB Phage Consultancy, LLC, Georgetown, Texas, USA; Queens College CUNY

## Abstract

Stenotrophomonas maltophilia is an opportunistic bacterium that is commonly associated with respiratory infections in immunocompromised patients, including cystic fibrosis patients. In this report, we introduce the complete genome sequence of S. maltophilia podophage Pepon, which is a T7-like phage closely related to the previously reported phage Ponderosa.

## ANNOUNCEMENT

Stenotrophomonas maltophilia is a Gram-negative opportunistic pathogen, and some strains found in natural environments and contaminated medical equipment have become multidrug resistant (1, 2). With its ability to cocolonize with Pseudomonas aeruginosa in cystic fibrosis patients, and increasing resistance to antibiotics, potential alternatives are being explored to treat S. maltophilia infections (1, 2). Here, we report the complete genome sequence of S. maltophilia phage Pepon, as part of the phage therapy development effort for controlling this pathogen.

Phage Pepon was isolated from an influent wastewater sample collected in September 2019 from the Texas A&M University wastewater treatment plant (College Station, TX) using S. maltophilia ATCC 17807 as its isolation and propagation host. Pepon was isolated using the double overlay method (3), and the host strain was cultured aerobically at 30°C in tryptone nutrient (0.5% tryptone, 0.25% yeast extract, 0.1% glucose, 0.85% NaCl, wt/vol) broth or agar. Phage genomic DNA was isolated from polyethylene glycol (PEG)-precipitated phage particles from ~8 mL phage lysate (>10^9^ PFU/mL) using a Promega Wizard DNA cleanup system as previously described (4), and the sequencing libraries were prepared as 300-bp inserts using a Swift 2S Turbo kit. The prepared sequencing libraries were sequenced on an Illumina MiSeq instrument with paired-end 150-bp reads using v2 300-cycle chemistry. The 348,904 raw sequence reads were quality controlled using FastQC v0.11.9 (www.bioinformatics.babraham.ac.uk/projects/fastqc) and then manually trimmed using FASTX-Toolkit v0.0.14 (http://hannonlab.cshl.edu/fastx_toolkit/) to result in 67,758 reads. A raw contig was assembled using SPAdes v3.50 (5) with 298-fold sequencing coverage. The genome was completed by PCR and Sanger sequencing of the product using the forward primer 5′-ATCCTGTCCTGTCAACCCCT-3′ and the reverse primer 5′-AACTGCGGCTTAGCAACTGA-3′. All annotations were carried out using the CPT Galaxy Apollo phage annotation platform (https://cpt.tamu.edu/galaxy-pub) (6–8). Structural annotations of Pepon were performed using MetaGeneAnnotator (9), Glimmer v3 (10), ARAGORN v2.36 (11), and tRNAscan-SE v2.0 (12). Gene function predictions were formed using InterProScan v5.48 (13), BLAST v2.9.0 (14), TMHMM v2.0 (15), HHpred (16), LipoP v1.0 (17), and SignalP v5.0 (18). All BLAST searches were conducted against the NCBI nonredundant and Swiss-Prot databases (19) with a maximum E value of 0.001. The genome-wide DNA sequence similarity was calculated using ProgressiveMauve v2.4 (20). All analyses were conducted with default settings.

**FIG 1:**
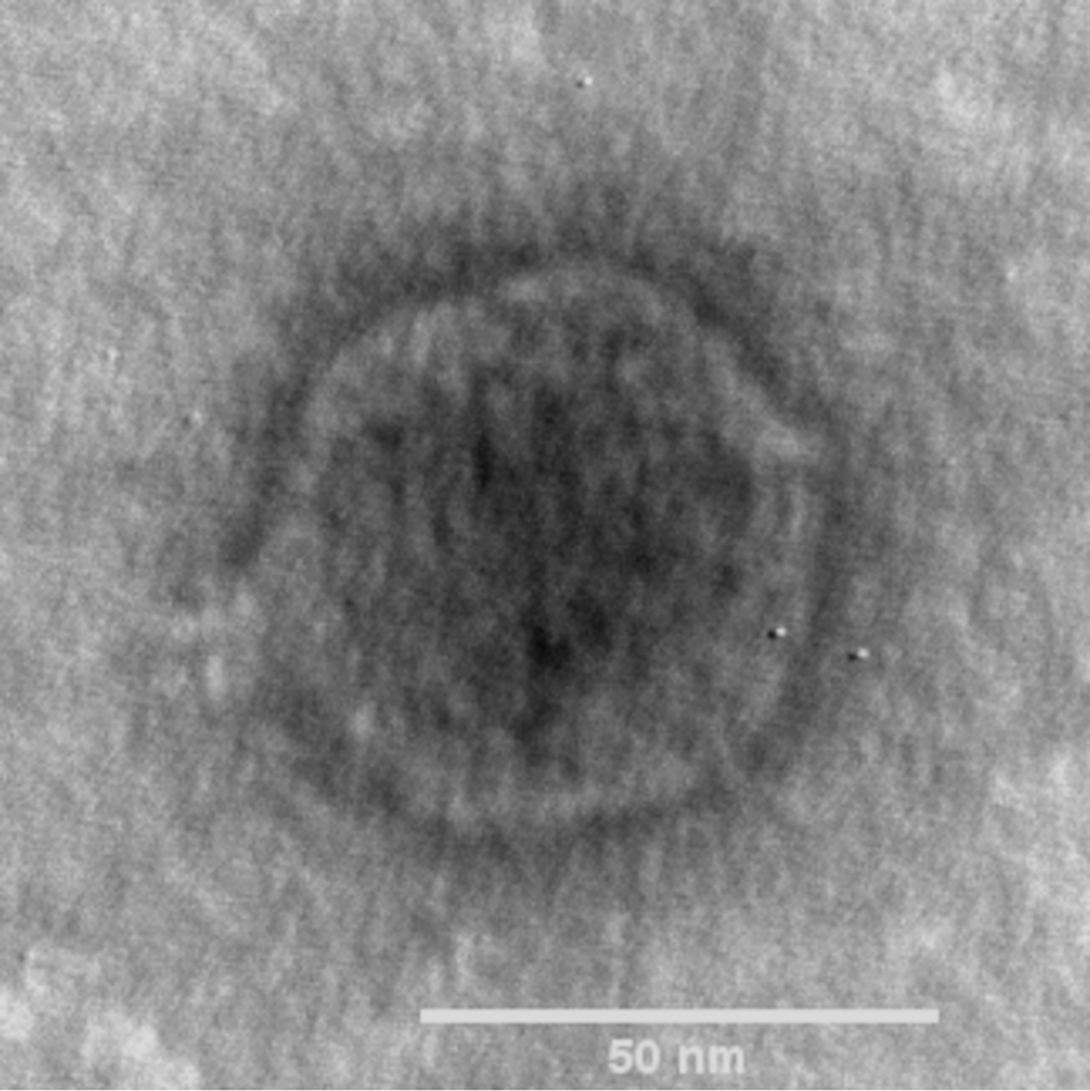
Transmission electron micrograph (TEM) of phage Pepon. Phage particles were diluted with TEM buffer (20 mM NaCl, 10 mM Tris-HCl [pH 7.5], 2 mM MgSO_4_) and captured on a freshly glow-discharged, Formvar carbon-coated grid. The grids were stained with 2% (wt/vol) uranyl acetate and observed on a JEOL 1200 EX TEM at 100 kV accelerating voltage at the Microscopy and Imaging Center at Texas A&M University.

Phage Pepon exhibited very large, clear plaques up to 8 mm in diameter with a 1-mm halo. The morphology of Pepon was confirmed as podophage (Fig. 1) by viewing the samples negatively stained with 2% (wt/vol) uranyl acetate via transmission electron microscopy at the Texas A&M Microscopy and Imaging Center.

The 42,532-bp genome of Pepon has a GC content of 60.0% and a coding density of 93.8%; it contains 53 protein-coding genes and no tRNAs. The genome termini were analyzed using PhageTerm (21), but the results were not conclusive. Phage Pepon shared the highest similarity with the S. maltophilia T7-like podophage Ponderosa (GenBank accession number MK903280) (22), with 90.6% genome-wide nucleotide identity, as determined using ProgressiveMauve, and 52 similar proteins (BLASTp; E value, <0.001). The predicted lysis cassette of Pepon appears to be bisected by the small and large terminases, with a class II holin upstream of the terminases and a downstream signal-arrest-release (SAR) endolysin and partially overlapping i-spanin/o-spanin.

### Data availability.

The Pepon genome sequence was deposited at GenBank under accession number MZ326858. The BioProject, SRA, and BioSample accession numbers are PRJNA222858, SRR14095254, and SAMN18509329, respectively.
